# The Effects of Obesity on Outcome in Preclinical Animal Models of Infection and Sepsis: A Systematic Review and Meta-Analysis

**DOI:** 10.1155/2020/1508764

**Published:** 2020-02-17

**Authors:** Wanying Xu, Dominique Pepper, Junfeng Sun, Judith Welsh, Xizhong Cui, Peter Q. Eichacker

**Affiliations:** ^1^Critical Care Medicine Department, NIH Clinical Center, National Institutes of Health, Bethesda, MD 20892, USA; ^2^National Institutes of Health Library, National Institutes of Health, Bethesda, MD 20892, USA

## Abstract

**Background:**

Clinical studies suggest obesity paradoxically increases survival during bacterial infection and sepsis but decreases it with influenza, but these studies are observational. By contrast, animal studies of obesity in infection can prospectively compare obese *versus* nonobese controls. We performed a systematic review and meta-analysis of animal investigations to further examine obesity's survival effect in infection and sepsis.

**Methods:**

Databases were searched for studies comparing survival in obese *versus* nonobese controls. We performed a systematic review and meta-analysis of animal investigations to further examine obesity's survival effect in infection and sepsis. *Methods*. Databases were searched for studies comparing survival in obese *versus* nonobese animals following bacteria, lipopolysaccharide, or influenza virus challenges.

**Results:**

Twenty-one studies (761 obese and 603 control animals) met the inclusion criteria. Obesity reduced survival in 19 studies (11 significantly) and the odds ratio (95% CI) of survival (0.21(0.13, 0.35); *I*^2^ = 64%, *p* < 0.01*p* < 0.01*p* < 0.01) but with high heterogeneity. Obesity reduced survival (1) consistently in both single-strain bacteria- and lipopolysaccharide-challenged studies (*n* = 6 studies, 0.21(0.13, 0.34); *I*^2^ = 64%, *p* < 0.01*p* < 0.01) but with high heterogeneity. Obesity reduced survival (1) consistently in both single-strain bacteria- and lipopolysaccharide-challenged studies (*n* = 6 studies, 0.21(0.13, 0.34); *I*^2^ = 64%, *p* < 0.01*p* < 0.01) but with high heterogeneity. Obesity reduced survival (1) consistently in both single-strain bacteria- and lipopolysaccharide-challenged studies (*n* = 6 studies, 0.21(0.13, 0.34); *I*^2^ = 64%, *p* < 0.01*p* < 0.01) but with high heterogeneity. Obesity reduced survival (1) consistently in both single-strain bacteria- and lipopolysaccharide-challenged studies (*n* = 6 studies, 0.21(0.13, 0.34); *I*^2^ = 64%, *p* < 0.01*p* < 0.01*p* < 0.01) but with high heterogeneity. Obesity reduced survival (1) consistently in both single-strain bacteria- and lipopolysaccharide-challenged studies (*n* = 6 studies, 0.21(0.13, 0.34); *I*^2^ = 31%, *p*=0.20 and *n* = 5, 0.22(0.13, 0.36); *I*^2^ = 0%, *p*=0.59, respectively), (2) not significantly with cecal ligation and puncture (*n* = 4, 0.72(0.08, 6.23); *I*^2^ = 75%, *p* < 0.01), and (3) significantly with influenza but with high heterogeneity (*n* = 6, 0.12(0.04, 0.34); *I*^2^ = 73%, *p* < 0.01). Obesity's survival effects did not differ significantly comparing the four challenge types (*p*=0.49). Animal models did not include antimicrobials or glycemic control and study quality was low.

**Conclusions:**

Preclinical and clinical studies together emphasize the need for prospective studies in patients accurately assessing obesity's impact on survival during severe infection.

## 1. Introduction

Obesity is a growing problem in the developed world and underlies chronic comorbidities that reduce overall life expectancy (e.g., hypertension and diabetes) [1–5]. While 25% or more of adults admitted to intensive care units (ICUs) in the United States and other developed countries are overweight or obese, whether this negatively impacts ICU outcomes is unclear [6–10]. This point is highlighted by conflicting data regarding obesity's effects on mortality in patients with bacterial infection and sepsis or viral influenza, two common reasons for ICU admission. Although several individual studies suggest that obesity worsens or has no impact on survival in patients with sepsis, two recent systematic reviews and meta-analyses both reported that overweight or obese body mass indices (BMIs) were paradoxically associated with improved outcomes in sepsis [11–15]. By contrast, two other systematic reviews and meta-analyses and a retrospective analysis of a large patient database found that for patients with influenza pneumonia, obesity increased the risk of either a combined endpoint of ICU admission and death or death alone [16, 17]. Another pooled analysis found that obesity increased the risk of death with influenza and pneumonia [18].

Research into the mechanisms and effects of obesity has relied on both diet- and genetically induced animal obesity models (e.g., leptin or leptin-receptor-deficient mice) [19]. These models have also been used to examine the impact of obesity on the host response and outcome in conditions associated with critical illness, including bacterial and viral infection [20–23]. In contrast to clinical studies which have been observational, retrospective, and possibly confounded by differing baseline characteristics and methodology, animal studies employ prospective controlled designs and uniform subjects that differ primarily in body weight and obesity cause. We therefore performed a systematic review and meta-analysis of animal studies to further examine how obesity may impact survival with bacterial, lipopolysaccharide, or influenza infection.

## 2. Methods

This systematic review was prepared using the PRISMA (Preferred Reporting Items for Systematic Reviews and Meta-Analyses) guidance for literature review and extraction of data, and a completed PRISMA checklist is provided in the Supplementary Material. Complete methods are provided in the Supplementary Material.

### 2.1. Literature Search and Study Selection

Using published guidelines [24] and search strategies presented in the Supplementary Material, two authors (DJP and PQE) identified relevant studies in the following databases from inception through January 25, 2017, and without language restrictions: PubMed, EMBASE, Scopus, and Web of Science. Included studies were searched for additional references. Studies were included if they compared survival in a nonobese control *versus* obese group in an animal model with bacterial infection, bacterial lipopolysaccharide (LPS), or influenza viral infection challenge. Studies without reported animal weights were included if they compared animals with a diet or genotype known to produce obesity.

### 2.2. Data Extracted and Outcomes Examined

Data were extracted by two authors (WZ and PQE) for each survival experiment in a report as described in the supplemental methods. The primary outcome examined was the effect of obesity on the odds ratio of survival based on the number of animals reported living at the end of observation periods. Group sizes, animal weights, fat masses, and blood glucose levels were determined as described in the supplemental methods. Secondary outcomes, presented in the supplemental methods, included the effect of obesity on organ injury based on physiologic or histologic measures; bacterial or viral clearance assessed by reported bacteria or viral counts in blood or tissue; and inflammatory cytokine and leptin levels in serum or tissue. Study quality and risk of bias were assessed in studies based on the Systemic Review Center for Laboratory animal Experimentation (SYRCLE) grading system and as previously described [25, 26]. Criteria for this grading are further outlined in the supplemental methods.

### 2.3. Statistical Analysis

The odds ratio of survival with obesity versus a nonobese control was estimated using a random-effects model [27]. In retrieved studies in which more than one experiment was performed using the same type of obesity model (i.e., diet or genetic), if the survival results of these experiments were qualitatively similar and consistent, these results were pooled to provide a single survival effect for the study. Experiments comparing two obese groups to a common control group or with similar survival in obese and control groups were analyzed as described in the supplemental methods. The effects of obesity on survival were analyzed based on the type of obesity model employed, the animal species studied, and the type of infectious or septic challenge employed. Heterogeneity among studies was assessed using the *Q* statistic and *I*^2^ value and was considered moderate or greater for *I*^2^ ≥ 35% [28]. Publication bias was to be assessed by funnel plot and Egger's regression if sufficient data were available. All analyses were performed using R (version 3.4.0) package *meta* (version 4.9-1) [29, 30]. Two-sided *p* values ≤0.05 were considered significant.

## 3. Results

**Figure 1:**
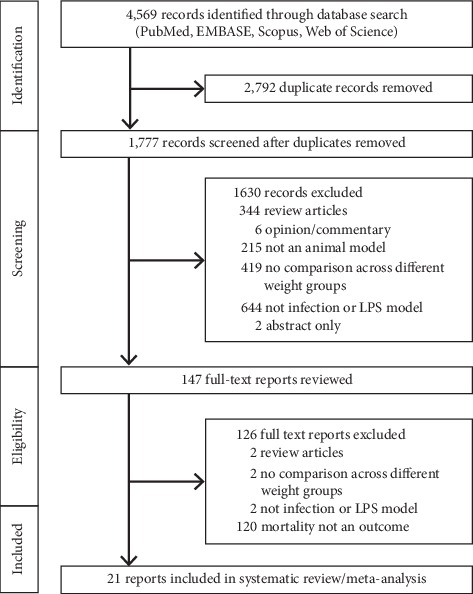
Flow diagram that summarizes the results of the literature search.

From 4,569 references identified in the literature search, 21 studies met inclusion criteria (Figure 1, Supplement References [Supplementary-material supplementary-material-1][Supplementary-material supplementary-material-1] in the Supplementary Material). These 21 studies included 52 experiments employing 603 control and 761 obese animals.  Table 1 provides details for each of the 52 experiments in the 21 studies regarding the animals and obesity and infection models employed.  Table 2 summarizes for each experiment the weight, fat mass, and glucose level recorded in control and obese groups and the total number of animals and the number of survivors in control and obese groups. Of these 21 studies, 12 included one or more experiments studying diet-induced obesity models alone, 5 included one or more experiments studying genetic-induced obesity models alone, and 4 included experiments, some of which studied genetic-induced obesity and others which studied diet-induced obesity. Eighteen and three studies employed mouse and rat models, respectively. Six studies employed a single-strain bacterial challenge, five an LPS challenge, four a cecal ligation and puncture (CLP) challenge, and six an influenza virus challenge.

### 3.1. Animal Weights, Fat Masses, and Baseline Blood Glucose Levels

In all 43 experiments providing data, the weight of animals employed was greater in obese compared to control groups (Table 2). In the 9 experiments not providing weight data, either the genotype or diet of obese animals is recognized to produce increased weight. Fat mass and blood glucose were greater in obese animals in the 11 and 12 experiments, respectively, providing data.

### 3.2. Effect of Obesity on Survival

In each study including more than one experiment in the same obesity model type (diet or genetic), the effects of obesity on the odds ratio of survival (95% CI) (OR) in individual experiments were never qualitatively different and heterogeneity for the combined ORs for these experiments was never significant (*p* ≥ 0.13 in four studies and *p* ≥ 0.12 in five studies in diet and genetic models, respectively) (Figures [Supplementary-material supplementary-material-1] and [Supplementary-material supplementary-material-1] in the Supplementary Material). The survival results of these experiments were pooled in subsequent analysis. In the four studies that examined both diet and genetic models of obesity, the results of these experiments were also pooled except when the effects of obesity type on survival were compared.

**Figure 2:**
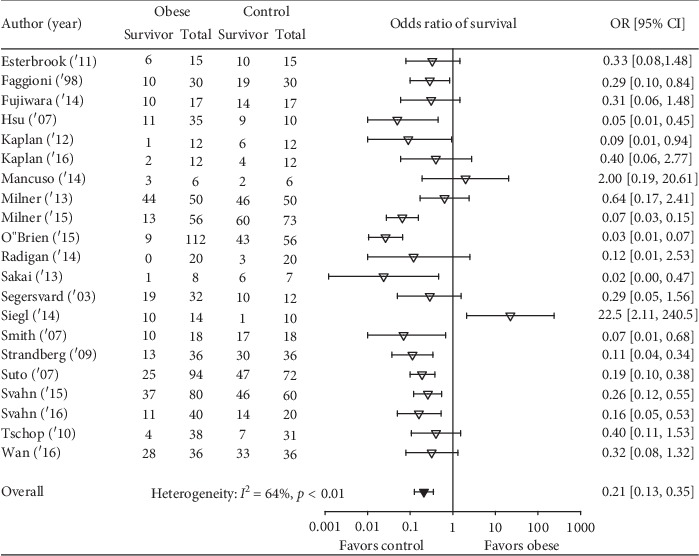
The number of total and surviving animals in obese and control groups for each of the 21 analyzed studies and the effects of obesity on the odds ratios (OR (95% CI)) of survival for each study. Also shown is the OR (95% CI) for the 21 studies and the associated *I*^2^ and its level of significance.

**Figure 3:**
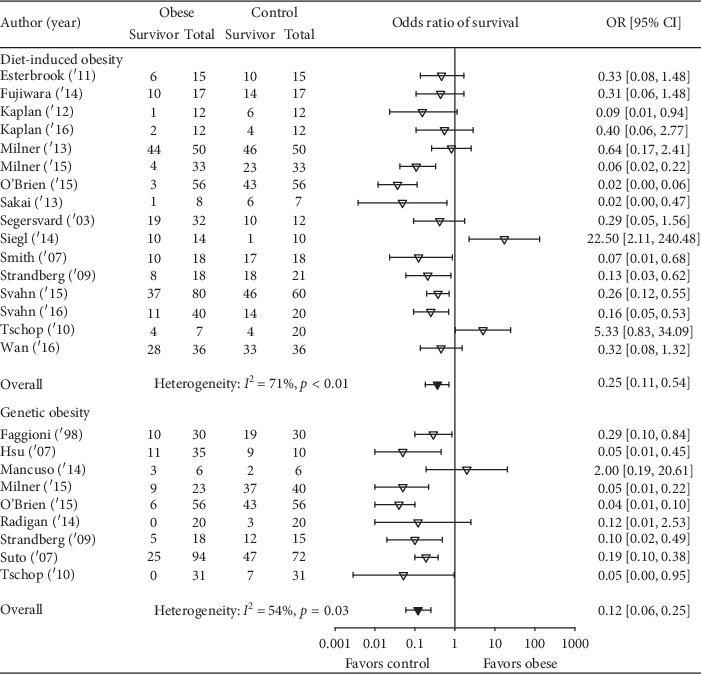
The number of total and surviving animals in obese and control groups for studies employing either a diet-induced obesity model or genetic-induced obesity model and the effects of obesity on the odds ratios (OR (95% CI)) of survival for each study and the overall OR (95% CI) for each type of obesity model and the associated *I*^2^ and its level of significance. As described in the results, because four studies examined both diet and genetic obesity models, this figure presents 25 comparisons, 16 with diet and 9 with genetic obesity models. The effects of obesity did not differ statistically significantly comparing the two types of models (*p*=0.19).

**Figure 4:**
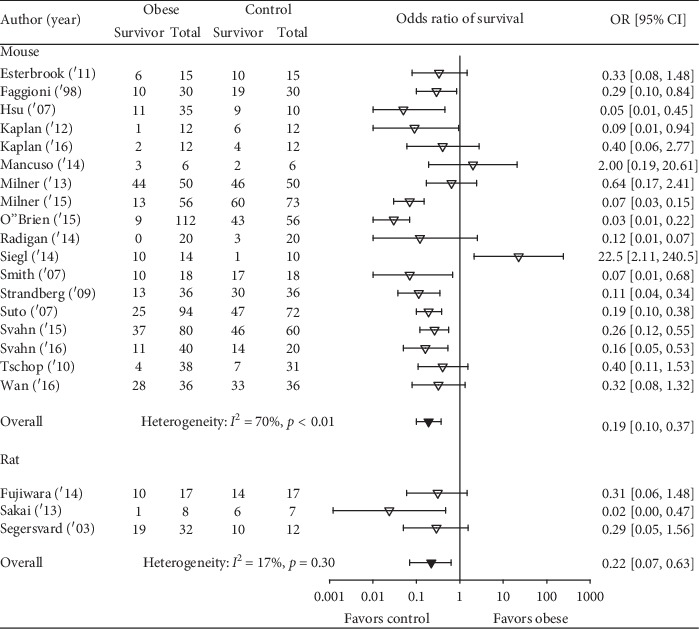
The number of total and surviving animals in obese and control groups for studies examining obesity in either mouse (18 studies) or rat (3 studies) and the effects of obesity on the odds ratios (OR (95% CI)) of survival for each study and the overall OR (95% CI) for each of the two species and the associated *I*^2^ and its level of significance. The effects of obesity did not differ statistically significantly comparing the two species (*p*=0.99).

**Figure 5:**
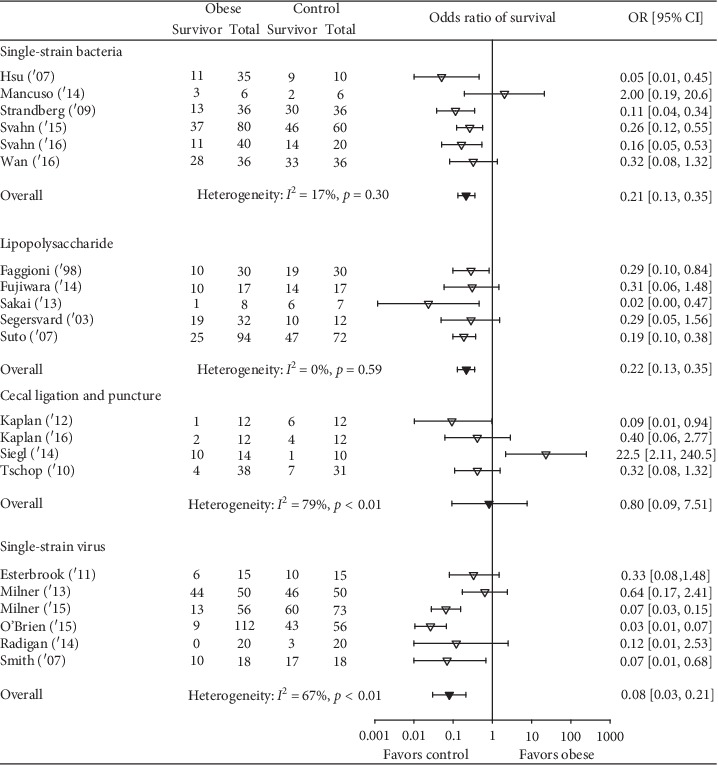
The number of total and surviving animals in obese and control groups for studies employing either a single strain bacterial infection model (*n* = 6 studies), a lipopolysaccharide model (*n* = 5 studies), a cecal ligation and puncture (polymicrobial infection) model (*n* = 4 studies), or a viral infection model (*n* = 6 studies) and the effects of obesity on the odds ratios (OR (95% CI)) of survival for each study and the overall OR (95% CI) for each type of infectious challenge model and the associated *I*^2^ and its level of significance. The effects of obesity did not differ significantly comparing the four model types (*p*=0.49).

Obesity was associated with reduced survival in 19 studies, and in 11 of these, the reductions were significant (Figure 2). Overall, obesity was associated with a reduced odds ratio (95% CI) of survival (0.21(0.13, 0.35); *I*^2^ = 64%, *p* < 0.01) but with moderate or more heterogeneity. The effect of obesity on the odds ratio of survival (95% CI) (OR) did not differ statistically significantly (*p*=0.19) comparing studies employing diet versus genetic obesity models (0.25 (0.11, 0.54); *I*^2^ = 71%, *p* < 0.01) versus (0.12 (0.06, 0.25); *I*^2^ = 54%, *p*=0.03) although there was moderate or more heterogeneity across each group of studies (Figure 3). This analysis examined 25 comparisons because 4 studies included experiments in both diet- and genetic-induced models. The effects of obesity on the OR also did not differ statistically (*p*=0.99) comparing studies including mice versus rats (0.22 (0.12, 0.39); *I*^2^ = 68%, *p* < 0.01 versus 0.22 (0.07, 0.63); *I*^2^ = 17%, *p*=0.30), but there was moderate or more heterogeneity across mouse but not the three rat studies (Figure 4). Finally, the effects of obesity on the OR did not differ significantly (*p*=0.49) comparing the four types of infectious or septic challenges (Figure 5). In studies with single-strain bacteria (*n* = 6) or LPS (*n* = 5) challenges, obesity reduced the ORs consistently (0.21(0.13, 0.34); *I*^2^ = 31%, *p*=0.20 and 0.22(0.13, 0.36); *I*^2^ = 0%, *p*=0.59). In CLP studies (*n* = 4), obesity reduced survival but not significantly (0.72(0.08, 6.23); *I*^2^ = 75%, *p* < 0.01). With influenza virus studies (*n* = 6), obesity reduced the OR in all studies (three significantly) but with moderate or more heterogeneity (0.12(0.04, 0.34); *I*^2^ = 73%, *p* < 0.01).

The slope (±SE) for the relationship between the ratio of obese to control animal weights versus the ln(OR) with obesity in individual experiments was consistent, with an increasing detrimental effect of obesity on survival with increasing weight ratio, but this was not significant (−0.81 (0.52), *p*=0.20). A funnel plot and Egger's regression (*p* = 0.06) suggested potential publication bias ([Supplementary-material supplementary-material-1] in the Supplemental Material). Only one experiment reported treating animals with an antimicrobial agent (Table 1) and none employed cardiopulmonary monitoring and support or glucose control as would be done in patients.

### 3.3. Effect of Obesity on Measures of Organ Injury and on Microbe, Cytokine, and Leptin Levels for the Different Infectious Challenges

The effect of obesity on measures of organ injury and on microbe, host inflammatory cytokine, and leptin levels was then examined in experiments providing data based on the type of infectious or septic challenge that was employed. For organ injury, with a single bacterial strain challenge, lung wet-to-dry weight ratios (*W*/*D*) were significantly increased with obesity at 24 h in one experiment and at 24 h and 96 h in another (Table 3). With CLP challenge, blood urea nitrogen (BUN) and/or alanine aminotransferase (ALT) as measures of kidney and liver injury, respectively, were significantly increased with obesity in two experiments at 6 or 24 h and a histologic lung injury score was increased at 6 h in another. With LPS, aspartate aminotransferase (AST) and liver histology score were increased with obesity at 6 h in one, but lung septal thickness and lung *W*/*D* at 6 h were not significantly different in two others. With influenza virus challenge, in six experiments, lung histology scores and/or alveolar lavage protein concentrations were significantly increased with obesity at various days from 3 to 8 days following challenge, but in two experiments, there were no significant changes in these parameters. Across all infectious challenges, in the 12 experiments reporting that obesity significantly affected organ injury, these were all increased.

In experiments reporting microbial data, with single-strain bacteria, obesity significantly increased blood and/or tissue bacteria counts at ≥48 h in five experiments and had no significant effect in five others ([Supplementary-material supplementary-material-1] in the Supplementary Material). With CLP, obesity significantly increased bacteria counts in blood and tissue at 24 h in one experiment and had no significant effect in another. With influenza, obesity significantly increased lung virus titers in two experiments 4 or 5 days after challenge but had no significant effect in 9 others ([Supplementary-material supplementary-material-1] in the Supplemental Material). In summary, in all eight experiments reporting that obesity significantly affected microbial levels, these were all increased.

[Supplementary-material supplementary-material-1] (for single bacteria strain, CLP, and LPS) and [Supplementary-material supplementary-material-1] (for influenza virus) in the Supplemental Material also summarize data for cytokine and leptin levels. When the effect of obesity on significant changes in cytokines in experiments was examined across the four types of infectious challenges: for TNF, eight experiments reported significant increases and five decreases; for IL-1b, four reported increases and one a decrease in blood but an increase in lung tissue; for IL-6, five reported increases and one a decrease; for IL-10, two reported increases and one a decrease; and for MIP-2a, three reported increases and none a decrease. Overall, inflammatory cytokines were reported to be significantly increased in 23 cases and only decreased in 8. For leptin levels, five experiments reported significant increases and two decreases.

### 3.4. Risk of Bias and Study Quality

Randomization, blinding, sample size calculations, and/or numbers of animals withdrawn from the study were not possible or described in studies. Therefore, the risk of bias was unclear or high in all studies examined and study quality was judged to be low ([Supplementary-material supplementary-material-1] in the Supplemental Material).

## 4. Discussion

This systematic review retrieved 21 studies that assessed the effect of a diet or genetic animal obesity model on survival following a single bacterial strain, CLP, LPS, or viral influenza challenge. In 19 studies, obesity was associated with reduced odds ratios of survival, 11 statistically significantly. The negative effect of obesity did not differ significantly comparing diet *versus* genetic models, mouse *versus* rat models, or the four types of infectious challenges. However, while there was moderate or high heterogeneity for the effects of obesity across the 21 studies, obesity's adverse effects were consistent across the 6 and 5 studies employing either a single strain of bacteria or an LPS challenge. Across all studies, the negative effect of obesity on survival increased with increasing weight ratio (i.e., obese to control group) but not significantly. Consistent with these adverse survival effects, in the 16 individual experiments presenting quantifiable data, obesity significantly increased some measure of organ injury in 12 experiments and no experiment reported a significant decrease.

Microbial clearance data, while limited, provide one possible basis for the decreased survival and increased organ injury seen with obesity in these models. Of the 23 experiments reporting data, obesity significantly increased blood or tissue bacteria or virus counts in 8 experiments and no experiment reported a decrease. Obesity-related insulin resistance and hyperglycemia or other changes may have impaired microbial clearance and worsened survival and organ injury [31–39]. Blood and tissue inflammatory cytokine levels including either TNF-*α*, IL-1b, IL-6, IL-10, or MIP-2a were also increased with obesity in 22 of 30 cases and may have contributed to inflammatory organ injury and worsened survival. Adipose tissue (fat mass), a source of inflammatory cytokine production, was increased in all reports presenting data, and obesity has previously been associated with a proinflammatory state [40–46].

Different from these findings in animal obesity models, two recent systematic reviews of observational clinical studies and a retrospective analysis of a large patient database found that obese body mass indices (BMI) appeared to increase survival in septic patients [13–15]. Preclinical and clinical results may differ for several reasons. Patients with infection and sepsis receive antimicrobial agents and other measures to clear the infectious nidus. Those with cardiopulmonary instability are aggressively supported. Blood glucose levels are also controlled clinically to counter hyperglycemia's adverse effects on microbial clearance. Such supportive measures may negate obesity's detrimental effects on microbial clearance or inflammatory mediator release while unmasking potentially protective effects. In contrast, only one of the animal experiments analyzed here administered an antimicrobial agent and none employed glucose control or organ support. Also, patients typically receive chronic treatment, such as glucose, cholesterol, and triglyceride control that would negate the long-term effects of obesity on the vascular dysfunction potentially worsening sepsis outcomes.

However, improved survival with obesity in clinical sepsis studies may be confounded by several factors. For comparably severe infection, obese patients may be admitted to the ICU more frequently where treatment is more comprehensive than in the non-ICU setting [13, 14]. Obese patients may also present with infection more amenable to treatment than nonobese patients. Antibiotics or hemodynamic support may favor obese patients in the acute period. For example, weight-based antibiotic dosing produces higher antibiotic concentrations at infection sites in obese patients while non-weight-based fluid administration could reduce the risk of fluid overload. Finally, the timing and technique for measurement of BMI may be unreliable in some clinical sepsis studies.

Different from clinical sepsis studies, but more consistent with the present findings in animal models, two systematic reviews and meta-analyses of influenza virus infection, with 6 studies in one and 22 in the other, suggested that obese BMIs are associated with worsened combined outcomes including mortality and the need for ICU admission and organ system support [16, 17]. While these analyses' combined endpoints confound their interpretation, in one which reported survival alone across three studies, obesity increased mortality significantly.

Two important questions these preclinical studies do not address are the following. First, how does obesity impact the outcome of septic patients who survive their initial course of acute infection and inflammation but progress to later sepsis with more chronic inflammation? Inflammation in these patients is characterized by both pro- and anti-inflammatory host responses. These patients frequently require prolonged invasive intensive care unit support, and maladaptive anti-inflammatory responses are believed to predispose them to secondary infection. However, clinical studies have not yet attempted to differentiate the impact of obesity on outcomes in acute versus chronically ill septic patients. Furthermore, animal models needed to test this question, although necessary, would be complex, requiring both an initial and follow-up septic challenges and prolonged observation. The second question has to do with whether comorbidities impact the outcome of sepsis and presumably then, whether obesity's impact on outcome in septic patients is influenced by these comorbidities. Comorbidities such as heart, lung, and kidney disease are all known to worsen the outcome from sepsis. Interestingly, though, a recent retrospective analysis of a large patient database which noted a protective effect of obesity in septic patients did not find that such comorbidities influenced these effects [15].

The findings from this analysis in combination with data from clinical studies point to several questions that should be addressed in future preclinical studies. Would the use of antimicrobial agents blunt or reverse the harmful survival effects of obesity in preclinical models? Related to that question, does dosing antimicrobial therapy based on weight increase its effectiveness on either microbial clearance or survival? Would blood glucose control alter the effect of obesity on microbial clearance and outcome in preclinical obesity models? Similarly, would cardiopulmonary support with or without antimicrobial therapy and glucose control reverse the harmful effects of obesity in preclinical models?

The present study has limitations. Organ injury and microbe and cytokine data were not provided in many reports which prevent firm conclusions regarding the basis for obesity's adverse effects in these preclinical studies. Weights were not reported for 9 studies and only 11 studies reported animal fat masses, although the obesity models employed are recognized to produce increases in each. Most studies did not include baseline data prior to the start of obese diets or infectious challenge. The literature search was conducted through January 2017, but it retrieved a relatively large number of reports included in the analysis and the overall survival findings were very similar across studies. Finally, the risk of bias was unclear or high across studies and study quality was judged to be low.

Determining whether obesity improves or worsens survival in critically ill patients with infection or sepsis is important. While the animal studies examined here support an adverse effect, some clinical data suggest the opposite. However as noted, in almost all cases, animal models lacked the types of support (e.g., antimicrobial therapy or glucose control) patients receive. If obesity is indeed protective during sepsis, understanding these beneficial effects might lead to new therapeutic approaches. But if obesity is detrimental for the acutely infected patient, then developing therapeutic approaches to counteract those harmful effects are necessary. These preclinical and clinical experiences together emphasize the need for prospective clinical studies that can accurately assess obesity's impact on survival during severe infection.

## 5. Conclusions

The results of the preclinical studies examined here are not consistent with the reported protective effects obesity has in retrospective, observational studies of patients with bacterial infection and sepsis but are consistent with obesity's reported harmful effects during influenza. These preclinical and clinical studies together emphasize the need for prospective studies in patients accurately assessing obesity's impact on survival during severe infection whether from a bacterial or viral influenza source.

## Figures and Tables

**Table 1:** Study characteristics.

Study (author, year)	Exp #	Species	Age (wk)	Sex	Obesity model		Challenge				Observation period
					Type	GT/DC	Type	Strain	Route	Dose	
*Single-strain bacteria models*											
Hsu, 2007	1	Mouse	8–12	F	Gen	ob/ob	*S. pneumoniae*	n/a	IT	10^5^ CFU	10 d
	2	Mouse	8–12	M	Gen	ob/ob/lep	*S. pneumoniae*	n/a	IT	10^5^ CFU	10 d
Strandberg, 2009	3	Mouse	5–7	M	Gen	ob/ob	*S. aureus*	n/a	IV	5 × 10^7^ CFU	17 d
	4	Mouse	5–7	M	DIO	HFD	*S. aureus*	n/a	IV	5 × 10^7^ CFU	17 d
Mancuso, 2014	5	Mouse	16–18	F	Gen	CPE^F/F^	*S. pneumoniae*	n/a	IT	5 × 10^4^ CFU	10 d
Svahn, 2015	6	Mouse	6	M	DIO	HFDP	*S. aureus*	n/a	IV	3.8–4.5 × 10^7^ CFU	17 d
	7	Mouse	6	M	DIO	HFDS	*S. aureus*	n/a	IV	3.8–4.5 × 10^7^ CFU	17 d
	8	Mouse	6	M	DIO	HFDS-HP	*S. aureus*	n/a	IV	3.8–4.5 × 10^7^ CFU	17 d
	9	Mouse	6	M	DIO	HFDS-LP	*S. aureus*	n/a	IV	3.8–4.5 × 10^7^ CFU	17 d
Svahn, 2016	10	Mouse	6	M	DIO	HFDS	*S. aureus*	n/a	IV	3–5.4 × 10^7^ CFU	17 d
	11	Mouse	6	M	DIO	HFDw6	*S. aureus*	n/a	IV	3–5.4 × 10^7^ CFU	17 d
Wan, 2016	12	Mouse	3-4	M	DIO	HFD	*E. coli*	n/a	IN	10^9^ CFU	96 h
	13	Mouse	3-4	M	DIO	HFD	*E. coli*	n/a	IN	10^10^ CFU	96 h
*Cecal ligation and puncture models*											
Tschop, 2010	14	Mouse	6–10	M	Gen	ob/ob	Polymicrobial	n/a	IP	n/a	240 h
	15	Mouse	6–10	M	DIO	HFD	Polymicrobial	n/a	IP	n/a	240 h
	16	Mouse	6–10	M	Gen	ob/ob	Polymicrobial	n/a	IP	n/a	240 h
Kaplan, 2012	17	Mouse	6	M	DIO	HFD	Polymicrobial	n/a	IP	n/a	30 h
Siegl, 2014	18	Mouse	19	M	DIO	HFD	Polymicrobial	n/a	IP	n/a	240 h
Kaplan, 2016	19	Mouse	6	M	DIO	HFD	Polymicrobial	n/a	IP	n/a	48 h
*Lipopolysaccharide*											
Fagioni, 1998	20	Mouse	5	F	Gen	ob/ob	*E. coli* 055:B5	n/a	IP	30 *μ*g	7 d
	21	Mouse	5	F	Gen	ob/ob	*E. coli* 055:B5	n/a	IP	100 *μ*g	7 d
	22	Mouse	5	F	Gen	ob/ob	*E. coli* 055:B5	n/a	IP	300 *μ*g	7 d
	23	Mouse	5	F	Gen	db/db	*E. coli* 055:B5	n/a	IP	30 *μ*g	7 d
	24	Mouse	5	F	Gen	db/db	*E. coli* 055:B5	n/a	IP	100 *μ*g	7 d
	25	Mouse	5	F	Gen	db/db	*E. coli* 055:B5	n/a	IP	300 *μ*g	7 d
Segersvard, 2003	26	Rat	NR	M	DIO	HFD35	*E. coli*	n/a	IP	2 mg	72 h
	27	Rat	NR	M	DIO	HFD60	*E. coli*	n/a	IP	2 mg	72 h
Suto, 2007	28	Mouse	NR	F	Gen	B6AY12w	*E. coli* 0111-B4	n/a	IP	50 *μ*g	7 d
	29	Mouse	NR	F	Gen	B6Ay12w	*E. coli* 0111-B4	n/a	IP	100 *μ*g	7 d
	30	Mouse	NR	F	Gen	B6-ob/ob	*E. coli* 0111-B4	n/a	IP	100 *μ*g	7 d
	31	Mouse	NR	F	Gen	B6Ay12w	*E. coli* 0111-B4	n/a	IP	200 *μ*g	7 d
	32	Mouse	NR	F	Gen	B6Ay10m	*E. coli* 0111-B4	n/a	IP	50 *μ*g	7 d
	33	Mouse	NR	F	Gen	B6Ay10m	*E. coli* 0111-B4	n/a	IP	100 *μ*g	7 d
Sakai, 2013	34	Rat	4	M	DIO	HFD	*E. coli* 0111-B4	n/a	IP	10 mg/kg	24 h
Fujiwara, 2014	35	Rat	4	M	DIO	HFD	*E. coli* 0111-B4	n/a	IP	10 mg/kg	12 h
	36	Rat	4	M	DIO	HFD	*E. coli* 0111-B4	n/a	IP	10 mg/kg	12 h
*Single-strain virus models*											
Smith, 2007	37	Mouse	NR	NR	DIO	HFD	H1N1 influenza A	A/PR8	IN	2 HG units	10 d
Easterbrook, 2011	38	Mouse	20	M	DIO	HFD	H1N1 influenza A	CA/09	IN	2.5 × 10^5^ pfu	15 d
	39	Mouse	20	M	DIO	HFD	H1N1 influenza A	NY312	IN	2.5 × 10^5^ pfu	15 d
	40	Mouse	20	M	DIO	HFD	H1N1 influenza A	Sw31	IN	50ul-SW31	15 d
Milner, 2013	41	Mouse	NR	M	DIO	HFD-UP	H1N1 influenza A	A/PR8	PO	5.3 × 10^5^ TCID50	13 d
	42	Mouse	NR	M	DIO	HFD-P	H1N1 influenza A	A/PR8	PO	5.3 × 10^5^TCID50	13 d
Radigan, 2014	43	Mouse	8–12	NR	Gen	db/db	H1N1 influenza A	A/WSN/33	IT	500 pu	14 d
	44	Mouse	8–12	NR	Gen	db/db	H1N1 influenza A	A/WSN/33	IT	1500 pu	14 d
O'Brien, 2015^*∗*^	45	Mouse	11	M	DIO	HFD	H1N1 influenza A	CA/09^*∗*^	IN	1 × 10^5^ TCID50	10 d
	46	Mouse	8	M	Gen	ob/ob	H1N1 influenza A	CA/09	IN	1 × 10^5^ TCID50	10 d
	47	Mouse	11	M	DIO	HFD	H3N2 influenza A	HK68	IN	6.3 × 10^5^ TCID50	10 d
	48	Mouse	8	M	Gen	ob/ob	H3N2 influenza A	HK68	IN	6.3 × 10^5^ TCID50	10 d
Milner, 2015	49	Mouse	14–16	M	DIO	HFD	H1N1 influenza A	CA/09	IN	5.8 × 10^5^	14 d
	50	Mouse	14–16	M	DIO	HFD	H1N1 influenza A	CA/09	IN	1.3 × 10^3^	14 d
	51	Mouse	13–16	M	Gen	LepR^H-/-^	H1N1 influenza A	CA/09	IN	5.8 × 10^5^	14 d
	52	Mouse	13–16	F	Gen	LepR^H-/-^	H1N1 influenza A	CA/09	IN	5.8 × 10^5^	14 d

B6-ob/ob: leptin-deficient mice; B6Ay10m and 12m: 10- and 12-week-old agouti peptide positive hyperphagic mice; CPE: lack functional carboxypeptidase enzyme; db/db: leptin receptor-deficient mice; DC: diet composition; DIO: diet-induced obesity; Exp: experiment; F: female; Gen: genetic-induced obesity; GT: genotype; HFD35: 35% of the energy from fat; HFD60: 60% of the energy from fat; HFD: high-fat diet; HFD-P: primed with virus; HFD-UP: unprimed; HFDP: polyunsaturated; HFDS-HP: high protein-to-carbohydrate ratio; HFDS-LP: low protein-to-carbohydrate ratio; HFDS: saturated; HFDw6: omega-6 fatty acid rich; IN: intranasal; IP: intraperitoneal; IT: intubation; IV: intravenous; LepR^H-/-^: lack leptin receptor signaling in hypothalamic neurons; M: male; n/a: not applicable; ob/ob: leptin-deficient mice; PO: oral administration; wk: week. ^*∗*^Oseltamivir treatment administered to animals.

**Table 2:** Survival, weight, fat mass, and glucose level table.

Study (author, year)	Exp #	Species	Obesity model		Weight (g)		Fat mass (g)		Glucose (mg/dl)		Control			Obese	
			Type	GT/DC	Control	Obese	Control	Obese	Control	Obese	Tot	Surv	Rep	Tot	Surv
*Single-strain bacteria models*															
Hsu, 2007	1	Mo	Gen	ob/ob	NR	NR	NR	NR	NR	NR	10	9	1	17	3
	2	Mo	Gen	ob/ob/lep	NR	NR	NR	NR	NR	NR	10	9	1	18	8
Strandberg, 2009	3	Mo	Gen	ob/ob	28.8 ± 0.5	39.3 ± 1.1	NR	NR	NR	NR	15	12	0	18	5
	4	Mo	DIO	HFD	28.8 ± 0.5	39.3 ± 1.1	5.5 ± 0.2	17.0 ± 0	NR	NR	21	18	0	18	8
Mancuso, 2014	5	Mo	Gen	CPE^F/F^	20 ± 0.5	47.5 ± 1	NR	NR^*∗*^	120 ± 5	180 ± 5	6	2	0	6	3
Svahn, 2015	6	Mo	DIO	HFDP	30 ± 1	35 ± 1	7.0 ± 0.5	14 ± 0.5	NR	NR	20	13	1	20	17
	7	Mo	DIO	HFDS	30 ± 1	45 ± 1	7.0 ± 0.5	20.0 ± 0.5	NR	NR	20	13	1	20	4
	8	Mo	DIO	HFDS-HP	30 ± 1	45 ± 1	14.05 ± 0.5	20.0 ± 0.5	NR	NR	20	16	0	20	8
	9	Mo	DIO	HFDS-LP	30 ± 1	45 ± 1	14.05 ± 0.5	20.0 ± 0.5	NR	NR	20	17	0	20	8
Svahn, 2016	10	Mo	DIO	HFDS	39 ± 1	45 ± 1	15 ± 0.5	20.0 ± 0.5	NR	NR	20	14	1	20	3
	11	Mo	DIO	HFDw6	39 ± 1	42.5 ± 1	15 ± 0.5	20.0 ± 0.5	NR	NR	20	14	1	20	8
Wan, 2016	12	Mo	DIO	HFD	37.5 ± 1	47.5 ± 1	NR	NR	225 ± 40	350 ± 25	18	18	0	18	18
	13	Mo	DIO	HFD	37.5 ± 1	47.5 ± 1	NR	NR	225 ± 40	350 ± 25	18	15	0	18	10
*Cecal ligation and puncture models*															
Tschop, 2010	14	Mo	Gen	ob/ob	NR	NR	NR	NR	NR	NR	20	4	1	20	0
	15	Mo	DIO	HFD	NR	NR	NR	NR	NR	NR	20	4	1	7	4
	16	Mo	Gen	ob/ob	NR	NR	NR	NR	NR	NR	11	3	0	11	0
Kaplan, 2012	17	Mo	DIO	HFD	23.4 ± 0.4	25.2 ± 0.4	MRI^*∗*^	MRI-inc^*∗*^	NR	NR	12	6	0	12	1
Siegl, 2014	18	Mo	DIO	HFD	27.7 ± 0.2	34.4 ± 0.5	NR	NR	NR	NR	10	1	0	14	10
Kaplan, 2016	19	Mo	DIO	HFD	27 ± 0.5	33 ± 1	2 ± 0.5	9.6 ± 2	165 ± 5	190 ± 5	12	4	0	12	2
*Lipopolysaccharide*															
Fagioni, 1998	20	Mo	Gen	ob/ob	20	40	NR	NR	100 ± 5	190 ± 5	5	5	0	5	3
	21	Mo	Gen	ob/ob	20	40	NR	NR	100 ± 5	190 ± 5	5	5	0	5	2
	22	Mo	Gen	ob/ob	20	40	NR	NR	100 ± 5	190 ± 5	5	4	0	5	0
	23	Mo	Gen	db/db	20	40	NR	NR	110 ± 5	310 ± 50	5	4	0	5	4
	24	Mo	Gen	db/db	20	40	NR	NR	110 ± 5	310 ± 50	5	1	0	5	1
	25	Mo	Gen	db/db	20	40	NR	NR	110 ± 5	310 ± 50	5	0	0	5	0
Segersvard, 2003	26	Ra	DIO	HFD35	415 ± 9	436 ± 8	3.4 ± 0.2^*∗∗*^	5.2 ± 0.2^*∗∗*^	NR	NR	12	10	1	16	9
	27	Ra	DIO	HFD60	415 ± 9	466 ± 5	3.4 ± 0.2^*∗∗*^	6.2 ± 0.2^*∗∗*^	NR	NR	12	10	1	16	10
Suto, 2007	28	Mo	Gen	B6AY12-	19.6	25.1	NR	NR	NR	NR	20	20	0	20	18
	29	Mo	Gen	B6Ay12-	19.6	25.1	NR	NR	NR	NR	20	17	1	20	7
	30	Mo	Gen	B6-ob/ob	19.6	52.8	NR	NR	NR	NR	20	17	1	24	0
	31	Mo	Gen	B6Ay12-	19.6	25.1	NR	NR	NR	NR	15	6	0	15	0
	32	Mo	Gen	B6Ay10m	19.6	51	NR	NR	NR	NR	6	2	0	5	0
	33	Mo	Gen	B6Ay10m	19.6	51	NR	NR	NR	NR	11	2	0	10	0
Sakai, 2013	34	Ra	DIO	HFD	289 ± 3.5	310.6 ± 1.9	3.2 ± 0.3^*∗∗*^	6.8 ± 0.3^*∗∗*^	102.0 ± 2.2	116 ± 3.7	7	6	0	8	1
Fujiwara, 2014	35	Ra	DIO	HFD	275 ± 1	294 ± 2.8	NR	NR	NR	NR	10	9	0	10	9
	36	Ra	DIO	HFD	450 ± 1	504.4 ± 7.3	NR	NR	NR	NR	7	5	0	7	1
*Single-strain virus models*															
Smith, 2007	37	Mo	DIO	HFD	NR	NR	NR	NR	86 ± 3	108 ± 9	18	17	0	18	10
Easterbrook, 2011	38	Mo	DIO	HFD	NR	NR	NR	NR	NR	NR	5	5	0	5	1
	39	Mo	DIO	HFD	NR	NR	NR	NR	NR	NR	5	5	0	5	5
	40	Mo	DIO	HFD	NR	NR	NR	NR	NR	NR	5	0	0	5	0
Milner, 2013	41	Mo	DIO	HFD-UP	30 ± 1	42.5 ± 1	NR	NR	NR	NR	4	0	0	4	0
	42	Mo	DIO	HFD-P	30 ± 1	42.5 ± 1	NR	NR	NR	NR	46	46	0	46	44
Radigan, 2014	43	Mo	Gen	db/db	20–25	30–35	NR	NR	NR	NR	10	3	0	10	0
	44	Mo	Gen	db/db	20–25	30–35	NR	NR	NR	NR	10	0	0	10	0
O'Brien, 2015	45	Mo	DIO	HFD	21	35	NR	NR	NR	NR	28	22	1	28	3
	46	Mo	Gen	ob/ob	21	53	NR	NR	NR	NR	28	22	1	28	6
	47	Mo	DIO	HFD	21	35	NR	NR	NR	NR	28	21	1	28	0
	48	Mo	Gen	ob/ob	21	53	NR	NR	NR	NR	28	21	1	28	0
Milner, 2015	49	Mo	DIO	HFD	30 ± 1	42.5 ± 1	NR	NR	70 ± 5	105 ± 5	21	21	0	21	4
	50	Mo	DIO	HFD	30 ± 1	42.5 ± 1	NR	NR	70 ± 5	105 ± 5	12	2	0	12	0
	51	Mo	Gen	LepR^H-/-^	30 ± 1	42.5 ± 1	NR	NR	NR	NR	20	18	0	10	5
	52	Mo	Gen	LepR^H-/-^	30 ± 1	42.5 ± 1	NR	NR	NR	NR	20	19	0	13	4

Exp: experiment; db/db: leptin receptor-deficient mice; DC: diet composition; DIO: diet-induced obesity; Gen: genetic; GT: genotype; HFD: high-fat diet; Mo: mouse; NR: not recorded; ob/ob: leptin-deficient mice; Ra: Rat; Rep: repeating control animals; Surv: number of animals surviving; Tot: total number of animals studied; wk: week. ^*∗*^Noted increased fat mass on MRI but not quantitated; ^*∗∗*^total fat mass weights calculated based on animal total weights (see methods).

**Table 3:** Effect of obesity compared to controls on parameters of organ injury.

Author (year)	Exp #	Model	Site of infection	Significant changes in organ injury comparing obese and nonobese groups	Overall effect of obesity on measure of organ injury
*Single-strain bacteria models*					
Wan '16	12	DIO	IN	Lung wet/dry ratio increased at 24 h with obesity	↑
	13	DIO	IN	Lung wet/dry ratio increased at 24 and 96 h with obesity	↑
*Cecal ligation and puncture models*					
Tschop '10	14	Gen	IP	BUN as a marker of renal injury increased at 24 h with obesity ALT as a marker of liver injury increased at 24 h with obesity	↑
Kaplan '12	17	DIO	IP	Histologic lung injury score increased at 6 h with obesity	↑
Kaplan '16	19	DIO	IP	ALT as a marker of liver injury increased at 6 h with obesity	↑
*Lipopolysaccharide*					
Sakai '13	34	DIO	IP	AST and liver histology score increased with obesity at 6 h	↑
Fujiwara ‘14	35	DIO	IP	No significant differences in lung septal thickness or *W*/*D* at 6 h	NSD
	36	DIO	IP	No significant differences in lung septal thickness or *W*/*D* at 6 h	NSD
*Single-strain virus models*					
Smith ‘07	37	DIO	IN	No significant difference in histologic lung injury score	NSD
Milner ‘13	42	DIO	PO	Histologic lung injury increased at 5 d and BAL protein increased at 5 d and 6 d with obesity	↑
Radigan ‘14	43	Gen	IT	BAL protein not significantly different at 4 d	NSD
	44	Gen	IT	BAL protein significantly increased at 4 d with obesity	↑
O'Brien	45	DIO	IN	Decreased lung epithelial regeneration and increased BAL albumin at 3 d and 6 d with obesity	↑
O'Brien	46	Gen	IN	Decreased lung epithelial regeneration and increased BAL albumin at 3 d and 6 d with obesity	↑
Milner ‘15	49	DIO	IN	BAL protein and albumin increased at 4 d and BAL protein increased at 8 d with obesity	↑
	51	Gen	IN	BAL protein increased at 8 d with obesity	↑

Exp: experiment; ALT: alanine aminotransferase; AST: aspartate aminotransferase; BAL: bronchoalveolar lavage; BUN: blood urea nitrogen; DIO: diet-induced obesity model; Gen: genetic model of obesity; NSD: no significant difference. ^*∗*^Remaining studies did not report organ injury data.

## Abbreviations

ALT: Alanine aminotransferase
AST: Aspartate aminotransferase
BMI: Body mass indices
BUN: Blood urea nitrogen
CLP: Cecal ligation and puncture
CI: Confidence interval
ICU: Intensive care units
IL: Interleukin
LPS: Lipopolysaccharide
MIP-2a: Macrophage inflammatory protein 2-alpha
OR: Odds ratio of survival
PRISMA: Preferred Reporting Items for Systematic Reviews and Meta-Analyses
SYRCLE: Systemic Review Center for Laboratory animal Experimentation
TNF: Tumor necrosis factor
*W*/*D*: Wet-to-dry weight ratios.

## Supplementary Materials

Supplementary Materials Supplementary Materials: search strategies and supplemental references showing the reports included in the analysis. Figure S1: this figure shows for each report including more than one experiment in the same diet-induced obesity model type the effects of obesity on the odds ratio of survival (95% CI) (OR) in individual experiments as well as the overall OR and its *I*^2^ and level of significance when these experiments were combined as described in the methods and results. The results of experiments in these comparisons were never qualitatively different and heterogeneity for overall ORs was not significant (*p* ≥ 0.13 in four reports with experiments which could be combined). Figure S2: this figure shows for each report including more than one experiment in the same genetic induced obesity model type the effects of obesity on the odds ratio of survival (95% CI) (OR) in individual experiments as well as the overall OR and its *I*^2^ and level of significance when these experiments were combined as described in the Methods and Results. The results of experiments in these comparisons were never qualitatively different and heterogeneity for overall ORs was not significant (*p* ≥ 0.12 in the five reports with experiments which could be combined). Figure S3: the funnel plot and Egger's statistic for the 25 studies described in the results. Table S1: effect of obesity compared to controls on bacteria counts and cytokine and leptin levels in bacteria-infected or LPS-challenged animals. Table S2: effect of obesity compared to controls on viral titer and cytokine and leptin levels in virus-infected animals. Table S3: summary of risk of bias scoring.
