# Supplementary material for: The Effects of Obesity on Outcome in Preclinical Animal Models of Infection and Sepsis: A Systematic Review and Meta-Analysis
Source: J Obes. 2020 Feb 17;2020:1508764. doi: 10.1155/2020/1508764 (PMC7053456; doi:10.1155/2020/1508764)

Supplemental Materials

Obesity Reduces Survival During Both Bacterial and Viral Influenza Infection in Preclinical Animal Models: A Systematic Review and Meta-Analysis

Wanying Xu [1], Dominique Pepper[1], Junfeng Sun [1], Judith Welsh [2],

Xizhong Cui [1], and Peter Q. Eichacker [1]

Critical Care Medicine Department [1] and National Institutes of Health Library [2]

Clinical Center, National Institutes of Health, Bethesda, MD 20892

Search strategies

PubMed
(obesity[majr] OR obesity[tiab] OR obese[tiab] OR diet, high fat[majr] OR "high fat"[tiab] OR leptin[majr] OR leptin[tiab] OR metabolic syndrome X[majr] OR "metabolic syndrome"[tiab]) AND (models, animal[mesh] OR "animal model"[tiab] OR "animal models"[tiab] OR animals, laboratory[mesh] OR "laboratory animal"[tiab] OR "laboratory animals"[tiab] OR "experimental animal"[tiab] OR "experimental animals"[tiab] OR "animal disease model"[tiab] OR "animal disease models"[tiab] OR mice[mesh] OR mouse[tiab] OR mice[tiab] OR murine[tiab] OR rats[mesh] OR rat[tiab] OR rats[tiab] OR rodent[tiab] OR rodents[tiab] OR swine[mesh] OR swine[tiab] OR pig[tiab] OR pigs[tiab] OR piglet[tiab] OR piglets[tiab] OR dogs[mesh] OR dog[tiab] OR dogs[tiab] OR canine[tiab] OR guinea pigs[mesh] OR "guinea pig"[tiab] OR "guinea pigs"[tiab] OR animals[mesh:noexp] OR animal[tiab] OR animals[tiab]) AND (sepsis[majr] OR sepsis[tiab] OR septic[tiab] OR bacteremia[majr] OR bacteremia[tiab] OR endotoxemia[majr] OR endotoxemia[tiab] OR endotoxins[majr] OR endotoxin[tiab] OR endotoxins[tiab] OR septicemia[majr] OR septicemia[tiab] OR fungemia[majr] OR fungemia[tiab] OR candidemia[majr] OR candidemia[tiab] OR lipopolysaccharides[majr] OR lipopolysaccharide[tiab] OR lipopolysaccharides[tiab] OR LPS[tiab] OR influenza[majr] OR influenza[tiab])

EMBASE #1
'obesity'/exp/mj OR obesity:ti,ab OR obese:ti,ab OR 'high fat':ti,ab OR 'leptin'/exp/mj OR leptin:ti,ab OR 'metabolic syndrome x'/exp/mj OR 'metabolic syndrome':ti,ab AND ('animal model'/exp OR 'animal model':ti,ab OR 'animal models':ti,ab OR 'laboratory animal':ti,ab OR 'laboratory animals':ti,ab OR 'experimental animal'/exp OR 'experimental animal':ti,ab OR 'experimental animals':ti,ab OR 'experimental organism'/exp OR 'animal disease model':ti,ab OR 'animal disease models':ti,ab OR 'mouse'/exp OR mouse:ti,ab OR mice:ti,ab OR murine:ti,ab OR 'rat'/exp OR rat:ti,ab OR rats:ti,ab OR rodent:ti,ab OR rodents:ti,ab OR 'pig'/exp OR pig:ti,ab OR pigs:ti,ab OR piglet:ti,ab OR piglets:ti,ab OR swine:ti,ab OR 'dog'/exp OR dog:ti,ab OR dogs:ti,ab OR canine:ti,ab OR 'guinea pig'/exp OR 'guinea pig':ti,ab OR 'guinea pigs':ti,ab OR animal:ti,ab OR animals:ti,ab) AND ('sepsis'/exp/mj OR sepsis:ti,ab OR septic:ti,ab OR 'bacteremia'/exp/mj OR bacteremia:ti,ab OR 'endotoxemia'/exp/mj OR endotoxemia:ti,ab OR 'endotoxin'/exp/mj OR endotoxin:ti,ab OR endotoxins:ti,ab OR 'septicemia'/exp/mj OR septicemia:ti,ab OR 'fungemia'/exp/mj OR fungemia:ti,ab OR 'candidemia'/exp/mj OR candidemia:ti,ab OR 'lipopolysaccharide'/exp/mj OR lipopolysaccharide:ti,ab OR lipopolysaccharides:ti,ab OR lps:ti,ab OR 'influenza'/exp/mj OR influenza:ti,ab) AND ([article]/lim OR [article in press]/lim OR [erratum]/lim OR [review]/lim)

EMBASE #2
'obesity'/exp/mj OR obesity:ti,ab OR obese:ti,ab OR 'high fat':ti,ab OR 'leptin'/exp/mj OR leptin:ti,ab OR 'metabolic syndrome x'/exp/mj OR 'metabolic syndrome':ti,ab AND ('sepsis'/exp/mj OR sepsis:ti,ab OR septic:ti,ab OR 'bacteremia'/exp/mj OR bacteremia:ti,ab OR 'endotoxemia'/exp/mj OR endotoxemia:ti,ab OR 'endotoxin'/exp/mj OR endotoxin:ti,ab OR endotoxins:ti,ab OR 'septicemia'/exp/mj OR septicemia:ti,ab OR 'fungemia'/exp/mj OR fungemia:ti,ab OR 'candidemia'/exp/mj OR candidemia:ti,ab OR 'lipopolysaccharide'/exp/mj

OR lipopolysaccharide:ti,ab OR lipopolysaccharides:ti,ab OR lps:ti,ab OR 'influenza'/exp/mj OR influenza:ti,ab) AND ('animal experiment'/de OR 'animal model'/de) AND ([article]/lim OR [article in press]/lim OR [erratum]/lim OR [review]/lim)

Scopus
(TITLE-ABS (obesity OR obese OR leptin OR "high fat" OR "metabolic syndrome") AND TITLE-ABS ("animal model" OR "animal models" OR "laboratory animal" OR "laboratory animals" OR "experimental animal" OR "experimental animals" OR "animal disease model" OR "animal disease models" OR mouse OR mice OR murine OR rat OR rats OR rodent OR rodents OR pi g OR pigs OR piglet OR piglets OR swine OR dog OR dogs OR canine OR "guinea pig" OR "guinea pigs" OR animal OR animals) AND TITLE-ABS (sepsis OR septic OR bacteremia OR endotoxemia OR endotoxin OR endotoxins OR septi cemia OR fungemia OR candidemia OR lipopolysaccharide OR lipopolysaccharides OR lp s OR influenza)) AND (limit-to (doctype , "ar") or limit-to (doctype , "re") or limit-to (doctype , "ip") or limit-to (doctype , "er"))

Web of Science
TITLE: (obesity OR obese OR leptin OR "high fat" OR "metabolic syndrome") AND TOPIC: ("animal model" OR "animal models" OR "laboratory animal" OR "laboratory animals" OR "experimental animal" OR "experimental animals" OR "animal disease model" OR "animal disease models" OR mouse OR mice OR murine OR rat OR rats OR rodent OR rodents OR pig OR pigs OR piglet OR piglets OR swine OR dog OR dogs OR canine OR "guinea pig" OR "guinea pigs" OR animal OR animals) AND TOPIC: (sepsis OR septic OR bacteremia OR endotoxemia OR endotoxin OR endotoxins OR septicemia OR fungemia OR candidemia OR lipopolysaccharide OR lipopolysaccharides OR lps OR influenza)
Refined by: DOCUMENT TYPES: (ARTICLE OR CORRECTION OR REVIEW)

Methods

Literature Search and Study Selection

Using published guidelines [23] and search strategies presented in S1 Text, two authors (DJP, PQE) identified relevant studies in the following databases from inception through January 25, 2017 and without language restrictions: PubMed, EMBASE, Scopus and Web of Science. Included studies were searched for additional references. Studies were systematically reviewed if they included experiments that employed an animal model examining the influence of obesity on survival with bacterial infection, bacterial lipopolysaccharide (LPS) or influenza viral infection challenge. Animal obesity models could be either diet- or genetically-induced. Studies not reporting the weight of animals were included if they compared animals with a diet or genotype known to produce obesity. Survival data had to be reported and compared in a non-obese control *versus* obese group.

Data Extracted and Outcomes Examined

For each experiment in a report comparing survival in obese and control groups, data was extracted by two authors (WZ and PQE) using a standardized tool including; number of animals in study groups: animal species and age of animals; diet composition or genotype of obesity model; animal weights, fat masses and blood glucose levels at the time of infectious challenge; strain or type and site, dose, and method of bacterial, LPS or viral challenge; use of antimicrobial or other supportive treatments; and observation period duration.

The primary outcome examined was the effect of obesity on the odds ratio of survival based on the number of animals reported living at the end of observation periods. In studies including more than one experiment employing the same obesity model, experiments were

compared and combined as described below in the *Statistical Analysis* section. When group sizes were reported as a range or survival was provided with Kaplan-Meier plots alone, study authors were contacted to obtain animal numbers contributing to groups and outcomes. If authors did not respond, group sizes were determined to be the average of the ranges presented and survival rates were estimated from Kaplan-Meier plots independently by two present authors (WZ and PQE) and agreed to by consensus. Animal weights, fat masses and blood glucose levels were estimated from figures providing data if not reported numerically.

Secondary-outcomes included the effect of obesity on: organ injury based on quantified physiologic or histologic measures; bacterial or viral clearance assessed by reported bacteria or viral counts in blood or tissue; and inflammatory cytokine and leptin levels in serum or tissue. Due to the differing assays and tissues sampled across studies, when summarizing aspects of organ injury, microbial clearance, and host cytokine responses, investigation results are presented as qualitative differences between obese versus control groups (i.e., increased or decreased) and whether these differences were statistically significant. For cytokines, data was collected regarding tumor necrosis factor (TNF), interleukins 1, 6 and 10 (IL-1, IL-6 and IL-10), and macrophage inhibitory protein-2 (MIP-2). Based on reported study designs, findings are recorded at 6h, 24h or ≥48h following bacteria and LPS challenges and daily for up to eight days following influenza challenge.

Study quality and risk of bias was assessed in studies based on the Systemic Review Centre for Laboratory animal Experimentation (SYRCLE) grading system and as previously described [24, 25]. In diet induced obesity models, study entry was judged to be at the time animals were initiated on their respective diets. In genetic induced obesity models, scoring regarding randomization and baseline characteristics were not considered applicable. Blinding of

outcome assessment was determined to be unclear if it was not reported whether animals were considered non-survivors based on moribund appearance or not. If moribund appearance was a basis for non-survivorship, outcome assessment was considered unblinded because obese and non-obese animals could be differentiated based on body habitus.

Statistical Analysis

The odds ratio of survival with obesity versus a non-obese control was estimated using a random-effects model [26]. In retrieved studies in which more than one experiment was performed using the same type of obesity model (i.e., diet or genetic), if the survival results of these experiment were qualitatively similar and consistent, these results were pooled to provide a single survival effect for the study. In five pairs of experiments in studies that compared two similar types of obesity models to a common control group, the two obese groups were pooled and compared to the common control group to avoid double counting the controls. In three pairs of experiments that compared one genetic and one diet induced obesity group to a common control group, the control group was employed twice in the separate analysis of the two obesity model types. But the two obese groups were pooled in combined analysis so the controls were only counted once. Experiments in which all controls and obese animals lived or died were only included in the analysis when pooled with other experiments in the same report. The effects of obesity on survival were analyzed based on: the type of obesity model employed, the animal species studied and the type of infectious or septic challenge employed. Heterogeneity among studies was assessed using the Q statistic and I^2 value and was considered moderate or greater for $I^2 \geq 35\%$ [27]. Publication bias was to be assessed by funnel plot and Egger's regression if

sufficient data was available. All analyses were performed using R (version 3.4.0) packages *meta* (version 4.9-1) [28, 29]. Two-sided p-values ≤ 0.05 were considered significant.

References to Supplemental Methods

1. Stroup DF, Berlin JA, Morton SC, Olkin I, Williamson GD, Rennie D, Moher D, Becker BJ, Sipe TA, Thacker SB: Meta-analysis of observational studies in epidemiology: a proposal for reporting. Meta-analysis Of Observational Studies in Epidemiology (MOOSE) group. *JAMA* 2000, 283(15):2008-2012.

2. Hooijmans CR, Rovers MM, de Vries RB, Leenaars M, Ritskes-Hoitinga M, Langendam MW: SYRCLE's risk of bias tool for animal studies. *BMC Med Res Methodol* 2014, 14:43.

3. Wever KE, Geessink FJ, Brouwer MAE, Tillema A, Ritskes-Hoitinga M: A systematic review of discomfort due to toe or ear clipping in laboratory rodents. *Lab Anim* 2017, 51(6):583-600.

4. DerSimonian R, Laird N: Meta-analysis in clinical trials. *Control Clin Trials* 1986, 7(3):177-188.

5. Higgins JP, Thompson SG: Quantifying heterogeneity in a meta-analysis. *Stat Med* 2002, 21(11):1539-1558.

6. R Foundation for Statistical Computing [https://www.r-project.org/]

7. meta: General package for meta-analysis. R package version. [https://CRAN.R-project.org/package=meta]

Supplemental References Showing the Reports Included in the Analysis

1. Hsu A, Aronoff DM, Phipps J, et al. Leptin improves pulmonary bacterial clearance and survival in *ob/ob* mice during pneumococcal pneumonia. Clinical Experimental Immunology 2007; 150: 332-339.
2. Strandberg L, Verdrengh M, Enge M, et al. Mice chronically fed high-fat diet have increased mortality and disturbed immune response in sepsis. PLOS one 2009; 4:e7605.
3. Mancuso P, O'Brien E, Prano J, et al. No impairment in host defense against *Streptococcus pneumoniae* in obese CPF$^{fat/fat}$ mice. PLOS one 2014; 9: e106420.
4. Svahn SL, Grahnemo L, Palsdottir V, et al. Dietary unsaturated fatty acids increase survival and decrease bacterial load during *Staphylococcus aureus* infection and improve neutrophil function in mice. Infect Immun 2015; 83: 514-521.
5. Svahn SL, Ulleryd MA, Grahnemo L, et al. Dietary omega-3 fatty acids increase survival and decrease bacterial load in mice subjected to *Staphylococcus aureus*-induced sepsis. Infect Immun 2016; 84: 1205-1213.
6. Wan T, Yuan G, Ren Y, et al. Diet-induced obese mice exhibit altered immune responses in acute lung injury induced by *Escherichia coli*. Obesity 2016; 24: 2101-2110.
7. Tschop J, Nogueiras R, Haas-Locvkie S, et al. CNS leptin action modulates immune response and survival in sepsis. J Neuroscience 2010; 30: 6036-6047.
8. Kaplan JM, Nowell M, Lahni P, et al. Short-term hhigh fat feeding increases organ injury and mortality after polymicrobial sepsis. Obesity 2012; 20: 1995-2002.
9. Siegl D, Annecke T, Johnson BL, et al. Obesity-induced hyperleptinemia improves survival and immune response in a murine model of sepsis. Anesthesiology 2104; 121: 98-114.

10. Kaplan JM, Nowell M, Lahni P, et al. Obesity enhances sepsis-induced liver inflammation and injury in mice. Obesity 2016; 7: 1480-1488.

11. Faggioni R, Fantuzzi G, Gabay C, et al. Leptin deficiency enhances sensitivity to endotoxin lethality. Am J Physiol. 1999; 45: R136-R142.

12. Segerssvard R, Sylvan M, Lempinen M, et al. Impact of chronic and acute high-fat feeding on acute experimental pancreatitis complicated by endotoxemia. Scand J Gastroenterol 2004; 39: 74-80.

13. Suto J. The A^y allele at the agouti locus enhances sensitivity to endotoxin-induced lethality in mice. Immunology 2007; 69: 931-937.

14. Sakaii S, Iizuka N, Fujiwara M, et al. Mild obesity reduces survival and adiponectin sensitivity in endotoxemic rats. J Surg Research 2013; 185: 353-363.

15. Fujiwara M, Miyi=oshi M, Sakai S, et al. Lard-based high-fat diet increases secretory leukocyte protease inhibitor expression and attenuates the inflammatory response of acute lung injury in endotoxemic rats. Clinical Nutrition 2014; 34: 997-1009.

16. Smith AG, Sheridan PA, Harp JB, et al. Diet-induced obese mice have increased mortality and altered immune responses when infected with influenza virus. J Nutr 2007; 137: 1236-1243.

17. Easterbrook JD, Dunfee RL, Schwartzman LM, et al. Obese mice have increased morbidity and mortality compared to non-obese mice during infection with the 2009 pandemic H1N1 influenza virus. Influenza and Other Respiratory Viruses 2011; 5: 418-425.

18. O'Brien KB, Vogel P, Duan S, et al. Impaired wound healing predisposes obese mice to severe influenza virus infection. J Infect Dis 2012; 205: 252-261.

19. Milner JJ, Sheridan PA, Karlsson EA, et al. Diet-induced obese mice exhibit altered heterologous immunity during a secondary 2009 pandemic H1N1 infection. J Immunol 2013; 191: 2472-2485.

20. Radigan KA, Morales-Nebreda L, Soberanes S, et al. Impaired clearance of influenza A virus in obese, leptin receptor deficient mice is independent of leptin signaling in the lung epithelium and macrophages. PLOS one 2014; 9: e108138.

21. Milner JJ, Rebeles J, Dhungana S, et al. Obesity increases mortality and modulates the lung metabolome during pandemic H1N1 influenza virus infection in mice. J Immunol 2015; 194: 4846-4859.

Table S1: Effect of obesity compared to controls on bacteria counts and cytokine and leptin levels in bacteria infected or LPS challenged animals

Author (year)	Exp #	Challenge	Model	Site of infection	Blood				Tissue				
					6h	24h	48h	>48h	Organ	6h	24h	48h	>48h
**Bacteria Counts**													
Hsu ('07)	1	SSB	ob/ob	IT			↑*		Lung			↑*	
	2	SSB	ob/ob/lep	IT			NSD					↑*	
Strandberg ('09)	3	SSB	ob/ob	IV		NSD			Kidney		NSD		↑*
Mancuso ('14)	5	SSB	CPE $^{F/F}$	IT					Lung<br>Spleen		NSD	NSD<br>NSD	
Svahn ('15)	6	SSB	HFDP	IV									↑*
	7	SSB	HFDS	IV									NSD
Svahn ('16)	10	SSB	HFDS	IV									NSD
	11	SSB	HFDS	IV									↑*
Wan ('16)	12	SSB	HFD	IN					Lung		NSD		NSD
	13	SSB	HFD	IN					Lung		NSD		NSD
Tschop ('10)	14	CLP	ob/ob	IP		↑*			PF		↑*		
Kaplan ('12)	17	CLP	HFD	IP		NSD			Lung<br>Liver<br>Spleen		NSD<br>NSD<br>NSD		
**TNF**													
Hsu ('07)	1	SSB	ob/ob	IT			↑*						
Strandberg ('09)	3	SSB	ob/ob	IV				NSD					
Mancuso ('14)	5	SSB	CPE $^{F/F}$	IT					Lung		↑*	NSD	
Svahn ('16)	10	SSB	HFDS	IV		NSD							
	11	SSB	HFDS	IV		NSD							
Wan ('16)	12	SSB	HFD	IN		↑*			Lung		↑*		
Kaplan ('12)	17	CLP	HFD	IP	↓*								
Siegl ('14)	18	CLP	HFD	IP		↓*	↓*	↓*	Lung		NSD	↓*	NSD
Kaplan ('16)	19	CLP	HFD	IP	↓*								
Fagioni '98	20	LPS	ob/ob	IP	NSD								

Fujiwara ‘14	35	LPS	HFD	IP					Lung<br>BALF	NSD	 NSD		
	36	LPS	HFD	IP					Lung<br>BALF		↓*<br>↓*		
**IL-1β**													
Strandberg ('09)	3	SSB	ob/ob	IV				↑*					
Mancuso ('14)	5	SSB	CPE $^{F/F}$	IT		NSD	NSD						
Svahn ('16)	10	SSB	HFDS	IV	NSD								
Siegl ('14)	18	CLP	HFD	IP	↓*	NSD	NSD		Lung	NSD	↑*	NSD	
Fagioni ‘98	20	LPS	ob/ob	IP	NSD								
**IL-6**													
Hsu ('07)	1	SSB	ob/ob	IT			↑*						
Strandberg ('09)	3	SSB	ob/ob	IV				NSD					
Mancuso ('14)	5	SSB	CPE $^{F/F}$	IT		NSD	NSD		Lung		NSD	NSD	
Svahn ('16)	10	SSB	HFDS	IV	NSD								
	11	SSB	HFDS	IV	NSD								
Wan ('16)	12	SSB	HFD	IN	↑*				Lung	↑*			
Tschop ('10)	14	CLP	ob/ob	IP	↓*								
Kaplan ('12)	17	CLP	HFD	IP	↑*								
Siegl ('14)	18	CLP	HFD	IP	NSD	NSD	NSD		Lung	NSD	NSD	↑*	
Kaplan ('16)	19	CLP	HFD	IP	NSD								
Fagioni ‘98	20	LPS	ob/ob	IP	NSD								
Segersvard ‘03	26	LPS	HFD35	IP				NSD					
Sakai ‘13	34	LPS	HFD	IP	NSD				Tissue	NSD			
**IL-10**													
Strandberg ('09)	3	SSB	ob/ob	IV				↑*					
Mancuso ('14)	5	SSB	CPE $^{F/F}$	IT					Lung		NSD	↓*	
Svahn ('16)	10	SSB	HFDS	IV	NSD								
	11	SSB	HFDS	IV	NSD								
Wan ('16)	12	SSB	HFD	IN	NSD				Lung	↑*			

Tschop ('10)	14	CLP	ob/ob	IP	NSD								
Segersvard '03	26	LPS	HFD35	IP				NSD					
Fujiwara '14	35	LPS	HFD	IP					Lung<br>BALF	NSD<br>NSD			
	36	LPS	HFD	IP					Lung<br>BALF	NSD<br>NSD			
**MIP-2**													
Hsu ('07)	1	SSB	ob/ob	IT			↑*						
Mancuso ('14)	5	SSB	CPE $^{F/F}$	IT					Lung		NSD	NSD	
Siegl ('14)	18	CLP	HFD	IP	↑*	NSD	NSD		Lung	↑*	↑*	NSD	
Fujiwara '14	35	LPS	HFD	IP					Lung<br>BALF	↑*<br>↑*			
	36	LPS	HFD	IP					Lung<br>BALF	↓*<br>NSD			
**Leptin**													
Hsu ('07)	1	SSB	ob/ob	IT			NSD		Lung			↓*	
Mancuso ('14)	5	SSB	CPE $^{F/F}$	IT	↑*								
Siegl ('14)	18	CLP	HFD	IP	↑*	↑*	NSD		Lung	↑*	NSD	↑*	
Kaplan ('16)	19	CLP	HFD	IP	↑*	↑*							

Exp – experiment; BALF- bronchoalveolar lavage fluid; CLP - cecal ligation and puncture; db/db - leptin receptor deficient mice; HFD - high fat diet; IN - intranasal; IP -Intraperitoneal; IT - intubation; IV -intravenous; LPS - lipopolysaccharide; NSD - no significant difference; ob/ob – leptin deficient mice; PF - peritoneal fluid; PO - oral administration; SSB - single strain bacterial

*significant data $p \leq 0.05$

Table S2: Effect of obesity compared to controls on viral titer, cytokine and leptin levels in virus infected animals

**Author (year)**	**Exp #**	**Model**	**Site of infection**	**Viral Titer**							
				**D1**	**D2**	**D3**	**D4**	**D5**	**D6**	**D7**	**D8**
**Viral Counts**											
Smith ('07)	37	HFD	IN			NSD			NSD		
Easterbrook ('11)	38	HFD	IN	NSD		NSD					
	39	HFD	IN	NSD		NSD					
	40	HFD	IN	NSD		NSD					
Milner ('13)	41	HFD	PO					↑*			
Radigan ('14)	43	db/db	IT		NSD		NSD				
	44	db/db	IT				↑*				
O’Brien ('15)	45	HFD	IN			NSD			NSD		
	46	ob/ob	IN			NSD			NSD		
Milner ('15)	50	HFD	IN				NSD				NSD
	51	HFD	IN				NSD				NSD
**TNF**											
Smith ('07)	37	HFD	IN	NSD		↓*			NSD		
Easterbrook ('11)	38	HFD	IN			↑*					
	39	HFD	IN			↑*					
	40	HFD	IN			↑*					
Radigan ('14)	43	db/db	IT		↑*						
	44	db/db	IT		↑*						
Milner ('15)	49	HFD	IN								NSD
	50	HFD	IN								NSD
	51	HFD	IN								NSD
	52	HFD	IN								NSD
**IL-1β**											
Smith ('07)	37	HFD	IN						NSD		
Easterbrook ('11)	38	HFD	IN			↑*					
	39	HFD	IN			↑*					

	40	HFD	IN			↑*					
Milner ('13)	41	HFD	PO					NSD			NSD
	42	HFD	PO					NSD			NSD
**IL-6**											
Smith ('07)	37	HFD	IN						↑*		
Radigan ('14)	43	db/db	IT		NSD						
	44	db/db	IT		NSD						
O'Brien ('15)	45	HFD	IN			NSD			NSD		
	46	ob/ob	IN			NSD			NSD		
	47	HFD	IN			NSD			NSD		
	48	ob/ob	IN			NSD			NSD		
**IL-10**											
Smith ('07)	37	HFD	IN						NSD		
Milner ('13)	41	HFD	PO					NSD			NSD
	42	HFD	PO					NSD			NSD
Milner ('15)	49	HFD	IN								NSD
	50	HFD	IN								NSD
	51	HFD	IN								NSD
	52	HFD	IN								NSD
**Leptin**											
Smith ('07)	37	HFD	IN	↑*		NSD			↑*		
Easterbrook ('11)	38	HFD	IN			↓*					
	39	HFD	IN			NSD					
	40	HFD	IN			NSD					
Milner ('15)	49	HFD	IN				↑*				

Exp – experiment; db/db - leptin receptor deficient mice; HFD - high fat diet; IN - intranasal; IT - intubation; NSD - no significant difference; ob/ob – leptin deficient mice; PO-oral administration

*significant data p≤0.05

Table S3. Summary of Risk of Bias Scoring

Study (Author y)	Any Randomization	Any Blinding	Sample size calculation	Random group allocation	Groups similar at baseline	Blinded group allocation	Random housing	Blinded interventions	Random outcome assessment	Blinded outcome assessment	Reported dropouts	Termination based on appearance	Other biases
**Single Strain Bacteria Models**													
Hsu '07	na	uc	no	na	na	na	uc	na	na	uc	no	uc	uc
Strandberg '09	uc	uc	no	uc	uc	uc	uc	uc	na	uc	no	uc	uc
Mancuso '14	na	uc	no	na	na	na	uc	na	na	no	no	yes	uc
Svahn '15	yes*	uc	no	uc	uc	uc	uc	uc	na	uc	no	uc	uc
Svahn '16	yes*	uc	no	uc	uc	uc	uc	uc	na	uc	no	uc	uc
Wan '16	uc	uc	no	uc	uc	uc	uc	uc	na	uc	no	uc	uc
**Cecal Ligation and Puncture Models**													
Tschop '10	uc	uc	no	uc	uc	uc	uc	**uc**	na	uc	no	uc	uc
Kaplan '12	yes*	uc	no	uc	uc	uc	uc	uc	na	uc	no	uc	uc
Siegl '14	uc	uc	no	uc	uc	uc	uc	uc	na	no	no	yes	uc
Kaplan '16	yes*	uc	no	uc	uc	uc	uc	uc	na	uc	no	uc	uc
**Lipopolysaccharide Models**													
Fagioni '98	na	uc	no	na	na	na	uc	na	na	uc	no	uc	uc
Segersvard '03	uc	uc	no	uc	uc	uc	uc	uc	na	uc	no	uc	uc
Suto '07	na	uc	no	na	na	na	uc	na	na	uc	no	uc	uc
Sakai '13	uc	uc	no	uc	uc	uc	uc	uc	na	uc	no	uc	uc
Fujiwara '14	yes*	uc	no	uc	uc	uc	uc	uc	na	uc	no	uc	uc
**Single Strain Virus Models**													
Smith '07	yes*	uc	no	uc	uc	uc	uc	uc	na	uc	no	uc	uc
Easterbrook '11	uc	uc	no	uc	uc	uc	uc	uc	na	uc	no	uc	uc
Milner '13	uc	uc	no	uc	uc	uc	uc	uc	na	uc	no	uc	uc
Radigan '14	uc	uc	no	na	na	na	uc	na	na	uc	no	uc	uc
O'Brien '15	uc	uc	no	uc	uc	uc	uc	uc	na	uc	no	uc	uc
Milner '15	uc	uc	no	uc	uc	uc	uc	uc	na	uc	no	uc	uc

na – not applicable; uc – unclear because there was no reporting as to whether the criteria was or wasn't met; * - these studies indicate animals were initially randomized but do not report a randomization method

Supplemental Figure 1

Author (year)- exp #	Obese Survivor	Obese Total	Control Survivor	Control Total	OR [95% CI]
Easterbrook ('11)-38	1	5	5	5	0.03 [0.00, 0.94]
Easterbrook ('11)-39	5	5	5	5	
Easterbrook ('11)-40	0	5	0	5	
Overall	Heterogeneity: n/a				0.03 [0.00, 0.94]
Fujuwara ('14)-35	9	10	9	10	1.00 [0.05, 18.57]
Fujuwara ('14)-36	1	7	5	7	0.07 [0.00, 0.97]
Overall	Heterogeneity: I^2 =44%, p=0.18				0.24 [0.02, 3.42]
Milner ('13)-41	0	4	0	4	
Milner ('13)-42	44	46	46	46	0.19 [0.01, 4.10]
Overall	Heterogeneity: n/a				0.19 [0.01, 4.10]
Milner ('15)-49	4	21	21	21	0.01 [0.00, 0.12]
Milner ('15)-50	0	12	2	12	0.17 [0.01; 3.90]
Overall	Heterogeneity: I^2 =56%, p=0.13				0.03 [0.00, 0.80]
O'brien ('15)-45	3	28	22	28	0.03 [0.01,0.15]
O'brien ('15)-47	0	28	21	28	0.01 [0.00, 0.11]
Overall	Heterogeneity: I^2 =0%, p=0.32				0.02 [0.01, 0.09]
Svahn ('15)-6,7	21	40	13	20	0.60 [0.20, 1.80]
Svahn ('15)-8	8	20	16	20	0.17 [0.04, 0.69]
Svahn ('15)-9	8	20	17	20	0.12 [0.03, 0.54]
Overall	Heterogeneity: I^2 =44%, p=0.17				0.25 [0.09, 0.71]
Wan ('16)-12	18	18	18	18	
Wan ('16)-13	10	18	15	18	0.25 [0.05, 1.18]
Overall	Heterogeneity: n/a				0.25 [0.05, 1.18]

Odds Ratio of Survival

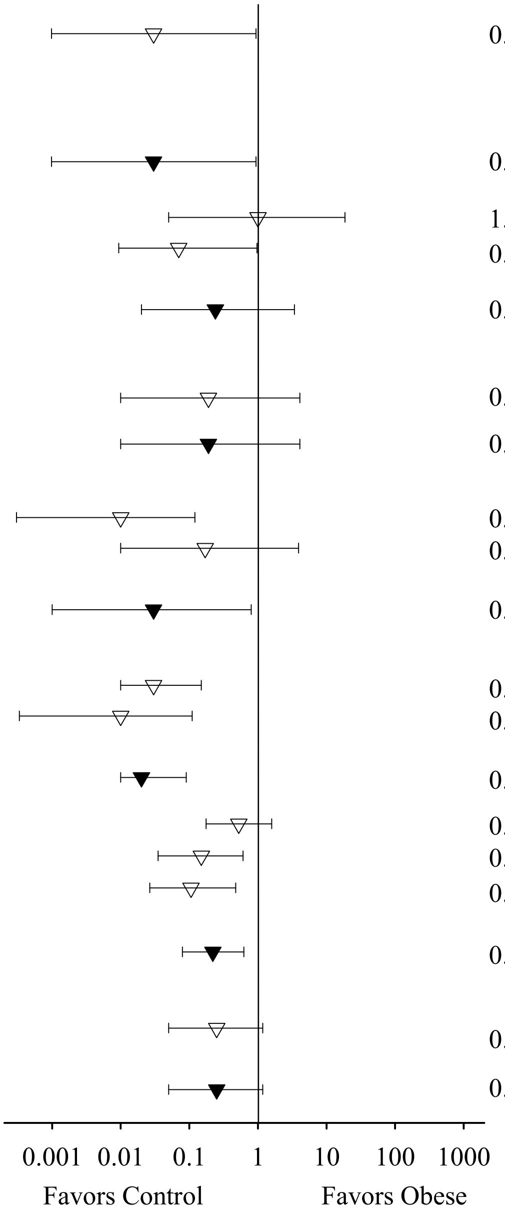

Supplemental Figure 2

Author (year)- exp #	Obese Survivor	Obese Total	Control Survivor	Control Total	OR [95% CI]
Faggioni ('98)-20	3	5	5	5	0.13 [0.00, 3.52]
Faggioni ('98)-21	2	5	5	5	0.06 [0.00, 1.79]
Faggioni ('98)-22	0	5	4	5	0.03 [0.00, 0.94]
Faggioni ('98)-23	4	5	4	5	1.00 [0.05, 22.18]
Faggioni ('98)-24	1	5	1	5	1.00 [0.05, 22.18]
Faggioni ('98)-25	0	5	0	5	
Overall	Heterogeneity: I^2 =0%, p=0.44				0.21 [0.05, 0.91]
Milner ('15)-51	5	10	18	20	0.11 [0.02, 0.75]
Milner ('15)-52	4	13	19	20	0.02 [0.00, 0.24]
Overall	Heterogeneity: I^2 =2%, p=0.31				0.06 [0.01, 0.26]
O'brien ('15)-46	6	28	22	28	0.07 [0.02, 0.27]
O'brien ('15)-48	0	28	21	28	0.01 [0.00, 0.11]
Overall	Heterogeneity: I^2 =58%, p=0.12				0.03 [0.00, 0.32]
Radigan ('14)-43	0	10	3	10	0.10 [0.00, 2.28]
Radigan ('14)-44	0	10	0	10	
Overall	Heterogeneity: n/a				0.10 [0.00, 2.28]
Suto ('07)-28	18	20	20	20	0.18 [0.01, 4.01]
Suto ('07)-29,30	7	44	17	20	0.03 [0.01, 0.15]
Suto ('07)-31	0	15	6	15	0.05 [0.00, 0.94]
Suto ('07)-32	0	5	2	6	0.16 [0.01, 4.36]
Suto ('07)-33	0	10	2	11	0.18 [0.01, 4.27]
Overall	Heterogeneity: I^2 =0%, p=0.75				0.06 [0.02, 0.18]
Tschop ('10)-14	0	20	4	20	0.09 [0.00, 1.78]
Tschop ('10)-16	0	11	3	11	0.11 [0.00, 2.33]
Overall	Heterogeneity: I^2 =0%, p=0.94				0.10 [0.01, 0.83]

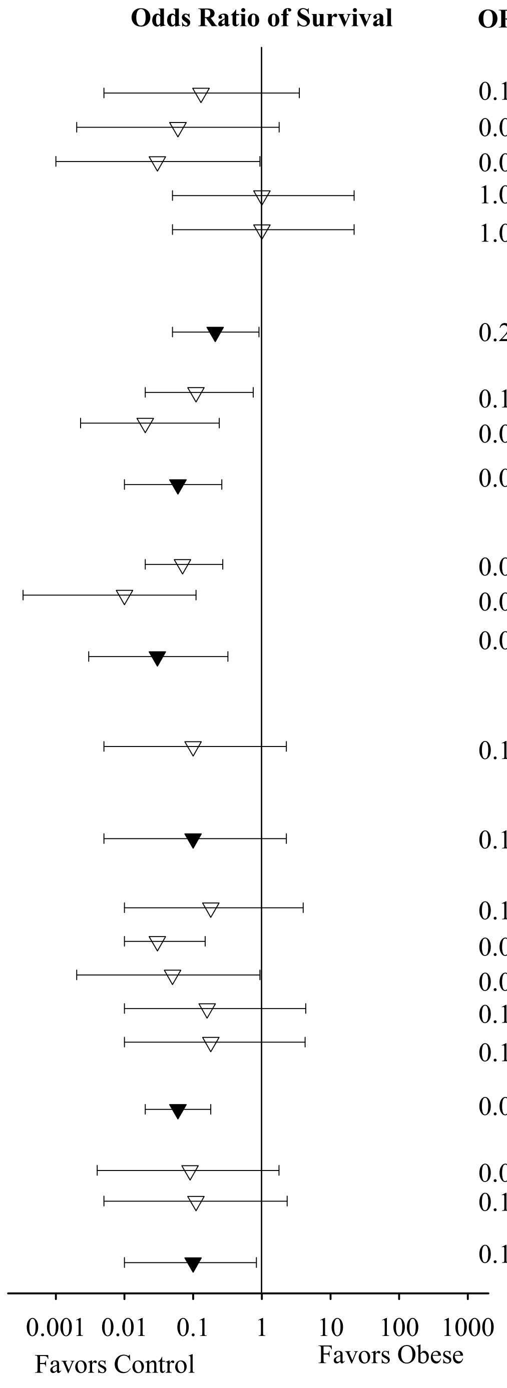

Supplemental Figure 3

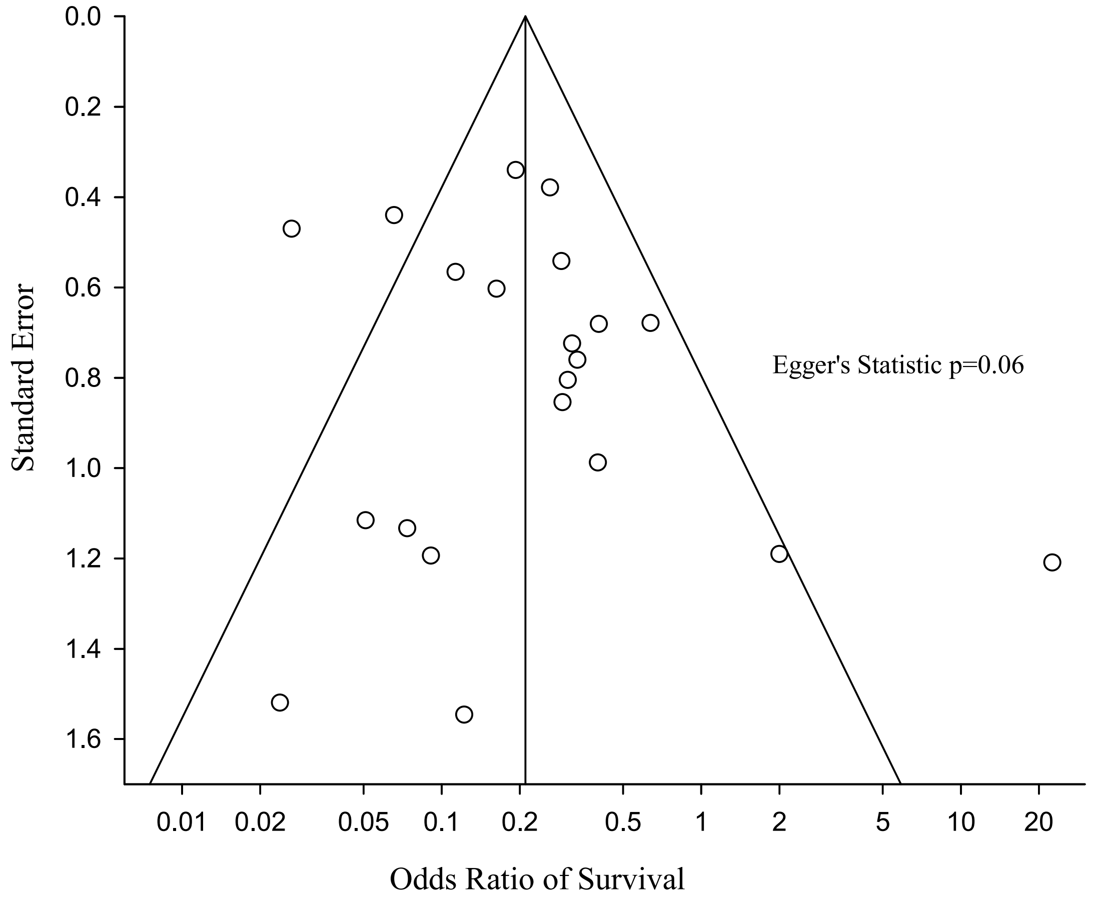

Supplement: Supplementary Materials — search strategies and supplemental references showing the reports included in the analysis. Figure S1: this figure shows for each report including more than one experiment in the same diet-induced obesity model type the effects of obesity on the odds ratio of survival (95% CI) (OR) in individual experiments as well as the overall OR and its I2 and level of significance when these experiments were combined as described in the methods and results. The results of experiments in these comparisons were never qualitatively different and heterogeneity for overall ORs was not significant (p ≥ 0.13 in four reports with experiments which could be combined). Figure S2: this figure shows for each report including more than one experiment in the same genetic induced obesity model type the effects of obesity on the odds ratio of survival (95% CI) (OR) in individual experiments as well as the overall OR and its I2 and level of significance when these experiments were combined as described in the Methods and Results. The results of experiments in these comparisons were never qualitatively different and heterogeneity for overall ORs was not significant (p ≥ 0.12 in the five reports with experiments which could be combined). Figure S3: the funnel plot and Egger's statistic for the 25 studies described in the results. Table S1: effect of obesity compared to controls on bacteria counts and cytokine and leptin levels in bacteria-infected or LPS-challenged animals. Table S2: effect of obesity compared to controls on viral titer and cytokine and leptin levels in virus-infected animals. Table S3: summary of risk of bias scoring. [file 1508764.f1.pdf]
